# First Evidence of Microplastics in Human Urine, a Preliminary Study of Intake in the Human Body

**DOI:** 10.3390/toxics11010040

**Published:** 2022-12-30

**Authors:** Concetta Pironti, Valentina Notarstefano, Maria Ricciardi, Oriana Motta, Elisabetta Giorgini, Luigi Montano

**Affiliations:** 1 Department of Medicine Surgery and Dentistry “Scuola Medica Salernitana”, University of Salerno, Via S. Allende, 84081 Baronissi, SA, Italy; 2 Department of Life and Environmental Sciences, DiSVA, Università Politecnica Delle Marche, 60121 Ancona, AN, Italy; 3 Department of Chemistry and Biology, University of Salerno, Via Giovanni Paolo II, 84084 Fisciano, SA, Italy; 4 Andrology Unit and Service of Lifestyle Medicine in UroAndrology, Local Health Authority (ASL) Salerno, Coordination Unit of the Network for Environmental and Reproductive Health (Eco-Food Fertility Project), “S. Francesco di Assisi Hospital”, 84020 Oliveto Citra, SA, Italy; 5 PhD Program in Evolutionary Biology and Ecology, University of Rome “Tor Vergata”, 00133 Rome, RM, Italy

**Keywords:** microplastic, urine, human body, Raman microspectroscopy

## Abstract

The ubiquitous presence of microplastics (MPs) and their health effects is a recent scientific topic. However, the investigation of MPs in human/biological matrices has several limitations due to analytical methods and sample treatment protocols. In this study, the presence of MPs in the urine samples of six volunteers from different cities in the south of Italy (three men and three women) was investigated by Raman microspectroscopy. The analysis pinpointed four pigmented microplastic fragments (4–15 μm size), with irregular shapes, which were characterized in terms of morphology and chemical composition. Polyethylene vinyl acetate (PVA), polyvinyl chloride (PVC), polypropylene (PP), and polyethylene (PE) MPs were found in four samples (PVA and PVC in one female sample and PP and PE in three male samples). This preliminary study suggests that MPs could pass through the gastrointestinal tract and are eliminated through biological processes.

## 1. Introduction

Plastic fragmentation represents an important source of environmental contamination. It is caused by chemical and physical ageing, related to high environmental temperatures, frictional forces, and UV exposure. Despite the important role of plastic in everyday life, such as in technical equipment and packaging, human exposure to microplastic contamination could have, over time, adverse health effects.

In the literature, the environmental presence of MPs has been widely investigated. In particular, MPs were detected in the air [1,2,3,4,5], in the soil [6,7,8], in the aquatic environments [9,10,11], including in marine species [12], and in several edible animal species (seafood and chicken) and food samples [13,14], such as salt, sugar, honey, milk, soft drink, and drinking water [15].

The risk to human health is a consequence of the release into the environment not only of micro- and nanoplastics, but also of additives, including unreacted monomers, organic and inorganic compounds, and all the other substances used in the formulation of plastic materials. Scientific studies have demonstrated the endocrine disruption activity and the carcinogenic properties of various additives, such as brominated flame retardants (BFRs), phenol derivatives (alkylphenols and bisphenol A), di(2-ethylhexyl)adipate (DEHA), and phthalic acid esters or phthalates (PAEs) [16,17]. Persistent organic pollutants (POPs), mainly polycyclic aromatic hydrocarbons (PAHs), polychlorinated biphenyls (PCBs), polybrominated diphenyl ethers (PBDEs), and dichlorodiphenyltrichloroethane (DDT), with negative effects on human health [18], can be adsorbed by MPs and transported in the environment [19]. Moreover, polystyrene (PS) nanoplastics themselves cause several undesirable effects, including a reduction in human lung cell viability, cell cycle arrest, the activation of inflammatory genes, and the promotion of cell apoptosis [20].

Today, the contamination of food and beverages by MPs is a great concern, even if plastics have been long considered inert after ingestion through the gastrointestinal (GI) tract. Recent studies have suggested that cell membranes and also the circulatory system could be crossed by small particles with sizes less than 10 μm [21]. In fact, MPs were found in human and pet animal stool specimens according to the previous assumption of the possibility to pass through the GI tract [22,23]. Stools collected from eight human volunteers (three men and five women, 33−65 years old) coming from several countries contained 20 pieces of MP per 10 g of stool. With regard to the polymer matrix, mainly polypropylene (PP) and polyethylene terephthalate (PET) sized 50−500 μm were found [23]. Concentrations of PET and polycarbonate (PC) MPs of up to 82,000 ng/g and 2100 ng/g were detected, respectively, in three meconium samples from six infants and ten adult feces samples [22]. Moreover, MPs have been found in the human placenta, human breastmilk, and sputum [24,25,26,27,28]. The presence of PP and PET microplastics was also detected in lung tissue samples (average of 1.42 ± 1.50 MP/g of tissue), supporting also inhalation as a route of exposure to environmental MPs [29].

Currently, there is a growing scientific interest in the presence of MPs in humans and their effects on the human body. After the recent identification of MPs in human fluids, in vitro and in vivo studies have been performed to analyze their adverse effects [30,31]. Moreover, the toxicological hazard due to human exposure to MPs has been evaluated by using polystyrene and polyethylene particles as a benchmark material for more complex microplastics [32].

In this light, in the present study, we analyzed human urine samples collected from six volunteers coming from different cities in the south of Italy. Raman microspectroscopy allowed us to detect, for the first time, the presence of microplastics in four out of six samples. This is a preliminary study to understand the evolution of MPs after the intake process in the human body and their possible accumulation.

## 2. Materials and Methods

### 2.1. Experimental Design

This study was a part of a comprehensive study on the influence of environmental conditions on human health, in particular, on young people (https://www.ecofoodfertility.it/, accessed on 12 July 2022). All methods were carried out in accordance with the Code of Ethics of the World Medical Association (Declaration of Helsinki) guidelines and regulations. All experimental protocols were approved by the Ethical Committee of the Local Health Authority Campania Sud-Salerno (Committee code n. 43 of 30 June 2015). Informed consent was obtained from recruited subjects before sample collection. To participate in this study, six selected consenting patients signed informed consent.

### 2.2. Inclusion/Exclusion Criteria

Participants recruited were high school and university people aged 16–35 years, living for at least 5 years in the considered areas. The selection was based on demographic data and lifestyle variables, western diet. The exclusion criteria are listed below:

✓ Body mass index <18.5 or >25; waist circumference >102 cm;
✓ Regular use of steroids or anabolic hormones (intake of dietary supplements or substances containing vegetal or animal extracts or trace elements);
✓ Tobacco smokers;
✓ Drug and alcohol users.

### 2.3. Sampling

A specific plastic-free protocol was adopted during the entire experiment to avoid contamination, in particular, glass sampling tubes were used to collect urine samples in a clean dedicated room.

### 2.4. Sample Digestion and Filtration

First, the organic components were removed from urine samples by applying a well-assessed digestion protocol set up and performed at the ARI Laboratory of Università Politecnica delle Marche (Ancona, Italy). For this purpose, KOH tablets (Sigma-Aldrich St. Louis, MO, USA) were added to 1.2 µm filtered deionized water, to obtain a 10% KOH solution. This solution was then added to urine samples at a ratio of 1:2 (*v*/*v*, sample/KOH). Flasks were sealed off with aluminum foils and let incubate for 48 h at 40 °C [24,25,33]. The obtained digestates were filtered by a vacuum pump connected to a filter funnel, through 1.2 µm pore-size filter membranes (Whatman GF/C, Buckinghamshire, United Kingdom). Finally, filter membranes were let dry at room temperature and stored in glass Petri dishes until Raman microspectroscopy analysis.

### 2.5. Quality Assurance and Control (QA/QC)

A plastic-free protocol, aimed at avoiding microplastic contamination, was adopted during sample collection, storage, processing, and analysis. Moreover, the digestion of urine samples, filtration, and RMS analysis steps were carried out in a dedicated room. All plastic tools were substituted with sterilized glass tools. Cotton laboratory coats and single-use latex gloves were worn during all phases of the experiment. Ethanol (70%) was also used to clean work surfaces before starting all procedures and during the experimental time. Glassware and instruments, such as scissors and tweezers, were washed using dishwashing liquid, triple-rinsed with 70% ethanol, and then rinsed with 1.2 µm filtered deionized water. All liquids, including 70% ethanol and deionized water, were filtered through 1.2 µm pore-size filter membranes (Whatman GF/A, Buckinghamshire, United Kingdom).

Furthermore, to assess the presence of contamination by MPs deriving from the laboratory environment and other external sources, environmental and procedural blanks were prepared and thoroughly analyzed. As regards the environmental blanks, a filter membrane soaked with 1.2 µm filtered deionized water was placed uncovered onto a Petri dish, and it was positioned in the dedicated room throughout the experiment. Moreover, a procedural blank with no sample was prepared following the same procedure as for samples together with every batch of samples. The filters deriving from environmental and procedural blanks were first inspected by a stereomicroscope.

### 2.6. Detection and Identification of MPs by Raman Microspectroscopy

Raman microspectroscopy (RMS) analysis was performed at the ARI Laboratory of Università Politecnica delle Marche (Ancona, Italy); an XploRA Nano Raman Microspectrometer (Horiba Scientific) was exploited. A first visible screening of all filter membranes, including those deriving from procedural blanks, was performed by a ×10 objective (Olympus MPLAN10×/0.25, Tokyo, Japan). Thus, the morphology of the detected MPs was characterized by a ×100 objective (Olympus MPLAN100×/0.90, Tokyo, Japan). Then, MPs were submitted to RMS analysis directly on the filter by using a 532 nm or 785 nm laser diode (spectral range 200–1800 cm^−1^; 600 lines per mm grating). Spectra were dispersed onto a 16-bit dynamic range Peltier-cooled CCD detector; before the spectral acquisition, the calibration of the spectrometer was performed on the 520.7 cm^−1^ line of silicon.

All raw Raman spectra were submitted to polynomial baseline correction and vector normalization, to decrease noise and improve spectrum quality (Labspec 6 software, Horiba Scientific). Finally, the Raman spectra collected for each MP were compared with specific spectral libraries of polymers and pigments (KnowItAll software, John Wiley & Sons, Inc., Hoboken, NJ, USA) to identify the polymer matrix [34,35]. Values of the Hit Quality Index (HQI) greater than 80 were considered satisfactory.

## 3. Results and Discussion

In the current study, for the first time, MPs were isolated from human urine samples. In particular, MPs were found in four of all the analyzed samples (in a female sample and in three male ones). MPs were characterized by RMS and classified in terms of shape, dimensions, color, polymer matrix, and pigments (Table 1).

In Table 2, the micro-images of the identified MPs are shown, together with the corresponding Raman spectra.

The micro-images allowed different colors to be distinguished for all the MPs, which were green, red, blue, and orange/yellow, irregular fragments with dimensions ranging from 4 µm to 15 µm. Moreover, the identified polymer matrices, such as polyethylene, polyvinyl chloride, and polypropylene, are currently reported as the most abundant in the environment [36,37].

A previous study analyzed the possibility of the absorption of MPs into the blood stream, and, thanks to fluorescence signature, the presence of these microparticles was identified in both blood and urine after tail vein injection, gavage, and pulmonary perfusion. This study was performed on mice and provided evidence that MPs can be absorbed and excreted through urine after 4 h of injection [38].

**Figure 1:**
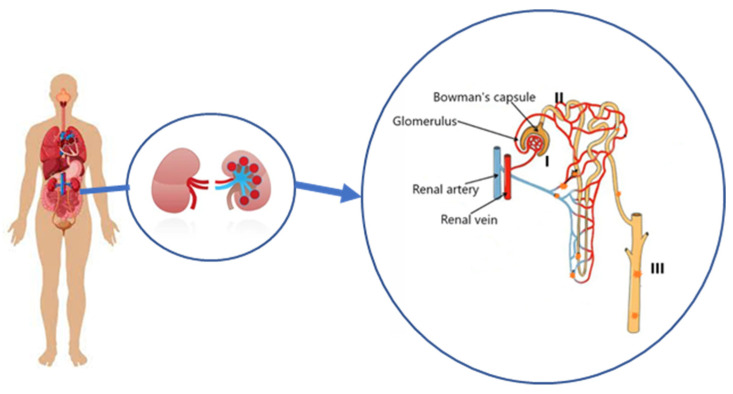
Description of the renal excretion of MPs: (I) through the bloodstream, MPs can flow inside of the glomerular tuft, without passing across the intact filtration barrier due to their size; (II) MPs are uptaken by the epithelial cells of the proximal convoluted tubules through endo- or macropinocytosis and then secreted into the tubular lumen; (III) MPs pass through the tubular system to be excreted with urine [39,40,41,42].

Kidney excretion could be a possible pathway by which to eliminate MPs, even if the glomerular filtration barrier is known to permit transportation only of particles with dimensions of ~10 nm [39,40]. On the other hand, as shown in Figure 1, it is possible that MPs pass through the renal tubule system: the mechanism could be related to exocytosis and endocytosis near the tubular epithelial cells after leaving the glomerulus via the efferent artery and entering the peritubular capillaries, before the excretion into the urine [41,42].

The distribution of MPs in the human body could be correlated to their length-to-diameter ratio: for example, the largest MPs (>0.2 μm) enter the cardiovascular system [43], while some smaller NPs (<0.1 μm) could stay in the blood [44,45]. Different physicochemical characteristics of particles, such as chemical properties, size, and activity, have been linked to the differences in their toxicity for years [46].

The environmental contamination of MPs is so extensive and widely known that the presence of particles within human fluids is not surprising. One of the potential sources of MPs could be associated with water contamination and the role of freshwater and potable water as a suspected potential source of MPs [47,48,49]. In the literature, the presence of fibers in freshwater has been reported. In particular, about 90% of MPs detected in drinking water were PE and PP, the same particles detected in our samples. The remaining 10% consisted of PET, PS, PVC, polyester (PES), and PA, the commercial materials used in food and cosmetic packaging [50]. Moreover, water samples taken at different positions within the drinking water supply chain (raw water and drinking water) have shown an average microplastic concentration of 700 particles/L (range 0–7000 particles/L) [49]. At the same time, MPs have also been found in soft drinks and milk. For example, in Mexican and German soft drinks, microplastic fragments and fibers, with various sizes (~0.1–3 mm) and colors, were detected [51,52]. Concerning the contamination of commercial milk, the source could be associated with poor a cleaning procedure for equipment, the surrounding environment, as well as water supply conditions and inadequate handling. However, the presence of MPs in human breast milk suggests the translocation of these particles to human fluids. Therefore, the presence of MPs in human urine could be the result of the combination and sum of several contributions from different contamination sources.

The present results are part of a preliminary study; in the future, it will be necessary to deeply evaluate the diffusion of MPs in a great number of subjects and to investigate the pathway of diffusion through the urinary system. However, these findings encourage the investigation of microplastics in human fluids, to assess the interaction and the effects of microplastics on the human body.

## 4. Conclusions

Environmental pollution and the specific accumulation of plastic microparticles increase stress on human safety and health. In this preliminary work, we evaluated for the first time the presence of MPs in urine samples collected from six volunteers from different cities in the south of Italy (three men and three women). Raman microspectroscopy was efficiently used to identify the polymeric matrices, allowing the detection of PVA, PVC, PP, and PE fragments (4–15 μm size). Recent studies related to the presence of microplastics in the environment have highlighted the contamination of water, food, and soil, and, hence, it is not surprising to detect these pollutants in human samples, too. In conclusion, we believe that the scientific research should focus on new methodologies and analytical protocols able to evaluate the effects of these contaminants in humans and animals, to better characterize the level of risk and to understand the possible transportation routes in biological fluids and tissues.

## Figures and Tables

**Table 1:** MPs presence in individual samples, including morphology, size, color, and polymer matrix.

Sample	N. of MPs	Shape	Size	Color	Polymer Matrix
#1 Female	0	-	-	-	-
#2 Female	2	irregular fragment	~15 µm	transparent	polyethylene vinyl acetate
		sphere	~7 µm	brown	polyvinyl chloride
#3 Female	0	-	-	-	-
#4 Male	3	irregular fragment	~5 µm	blue	polypropylene
		irregular fragment	~10 µm	blue/grey	polypropylene
		irregular fragment	~15 µm	green	polypropylene
#5 Male	1	irregular fragment	~4 µm	red	polyethylene
#6 Male	1	irregular fragment	~10 µm	green	polypropylene

**Table 2:** Micro-images of the identified MPs and corresponding Raman spectra.

Sample	Micro-Images	Raman Spectrum and Identified Polymer Matrix
#2	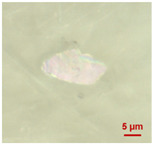	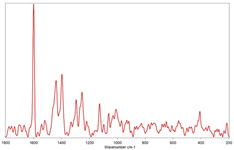 polyethylene vinyl acetate
	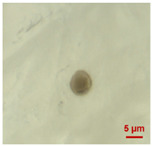	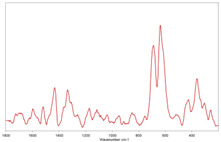 polyvinyl chloride
#4	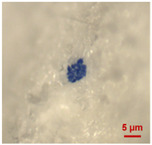	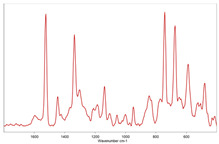 polypropylene
	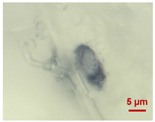	
	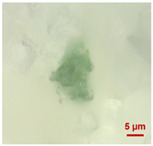	
#5	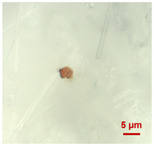	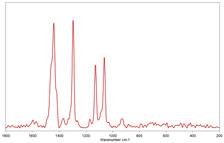 polyethylene
#6	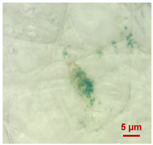	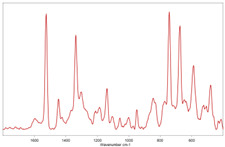 polypropylene

## Data Availability

The data presented in this study are available on request from the corresponding author.
